# A novel application of Gini coefficient for the quantitative measurement of bacterial aggregation

**DOI:** 10.1038/s41598-019-55567-z

**Published:** 2019-12-12

**Authors:** Yu-ming Cai, David S. Chatelet, Robert P. Howlin, Zhi-zhong Wang, Jeremy S. Webb

**Affiliations:** 1 National Biofilms Innovation Centre, Institute of Life Sciences, University of Southampton, Southampton, SO17 1BJ UK; 2 Biomedical Imaging Unit, University of Southampton, Southampton, SO16 6YD UK; 3 NIHR Southampton Respiratory Biomedical Research Centre, Southampton, SO16 6YD UK; 4 School of Biomedical Engineering, Shanghai Jiao Tong University, 800 Dongchuan Rd, Minhang District, Shanghai, China

**Keywords:** Computational biology and bioinformatics, Microbiology

## Abstract

Non-surface attached bacterial aggregates are frequently found in clinical settings associated with chronic infections. Current methods quantifying the extent to which a suspended bacterial population is aggregated mainly rely on: (1) cell size distribution curves that are difficult to be compared numerically among large-scale samples; (2) the average size/proportion of aggregates in a population that do not specify the aggregation patterns. Here we introduce a novel application of Gini coefficient, herein named Aggregation Coefficient (AC), to quantify the aggregation levels of cystic fibrosis *Pseudomonas aeruginosa* (CF-PA) isolates *in vitro* using 3D micrographs, Fiji and MATLAB. Different aggregation patterns of five strains were compared statistically using the numerical AC indexes, which correlated well with the size distribution curves plotted by different biovolumes of aggregates. To test the sensitivity of AC, aggregates of the same strains were treated with nitric oxide (NO), a dispersal agent that reduces the biomass of surface attached biofilms. Strains unresponsive to NO were reflected by comparable AC indexes, while those undergoing dispersal showed a significant reduction in AC index, mirroring the changes in average aggregate sizes and proportions. Therefore, AC provides simpler and more descriptive numerical outputs for measuring different aggregation patterns compared to current approaches.

## Introduction

Biofilms have been reported to grow in manifestations as non-attached floating bacterial aggregates and surface attached communities. To distinguish these two lifestyles in this study, we refer to ‘biofilm’ when describing surface attached communities and ‘aggregate’ to describe suspended ones. Whilst attached biofilms formed by a spectrum of bacterial strains have been extensively studied *in vitro*, the regulatory mechanisms of suspended aggregates are far less understood. However, aggregates are frequently found in clinical and environmental settings^1–6^. Some clinical isolates such as *Pseudomonas aeruginosa*, *Staphylococcus aureus* and *Stenotrophpmonas maltophilia* cultured from chronic cystic fibrosis patients were reported to show impaired biofilm formation *in vitro*, which might be caused by genetic evolution within the host^6^. In contrast, most bacteria in CF airways and wounds are found suspended in secretions, with their morphologies differing significantly from surface attached biofilms in size and structure^1,7,8^. Moreover, a study showed that biofilm-defective *P. aeruginosa* mutant strains impaired in surface attached biofilm formation could still form aggregates in gels and exhibit high tolerance to antibiotics^9^. Hence, focused studies on suspended CF-PA aggregates is critically need due to their potentially different regulatory mechanism compared to biofilm.

Previous reports demonstrated that disruption of the aggregates by mechanical disturbance (sonication and vortex) improved antibiotic treatments, suggesting physical properties play an important role in the antibiotic tolerance^2,10^. As such, it is important to quantify and describe the properties of aggregates for treatment and analysis. Whilst software such as COMSTAT^11^ analysing micrographs have been extensively applied for the quantification of biofilms, so far, the description of bacterial aggregate properties mainly relies on the visual description of microscopic images^12^; average size/volume of aggregates measured in micrographs^13–15^; or individual particle measurement using specific equipment such as laser-diffraction particle-size scanning analysis (LDA)^16^ or flow cytometry^17^. Bar graphs and fold lines are frequently adopted to represent the percentages of aggregates and aggregate sizes distribution^18^. Whilst the comparisons of size/volume distribution curves/bars can show the details of a population, it is difficult to obtain quantifiable results from multiple graphs. Moreover, when comparing multi-species aggregates from one sample or single-species aggregates from different samples, the sizes of cells differ, making the interpretation of results difficult. The average sizes or total percentages of aggregates for different cultures can be easily compared numerically, but the aggregates are subjectively defined by size thresholds. Whether it is a few larger aggregates or many smaller aggregates, i.e, how aggregated the population is, cannot be clarified by the mean sizes or percentages *per se*.

The underlying situation of bacterial grouping in a population is conceptually similar to the theory of the Gini coefficient, which is frequently applied in economics to measure the income inequality in a group of residents^19^. The more equal the distribution, the lower the index, with 0 for a perfect equality and 1 for a perfect inequality. The Gini coefficient has been widely applied in different areas, such as the unequal yield and population distribution^20,21^, valuation of disease detection efficiency^22^ and gene expression profiles^23,24^. As for bacterial communities, the most frequent usage of Gini coefficient is for bacteria diversity and species distribution in different environmental settings and biofilm communities^25,26^. However, this principle has not been reported to describe the unequal size distribution of aggregates within planktonic or biofilm cultures. How different strains adopt their grouping patterns in the same environment, or how the same strain adopts different grouping patterns under different environments, might be related to their survival mechanism in response to stresses such as nutrient depletion^16^. Furthermore, aggregates are usually more difficult to treat compared to planktonic single cells. Thus, a simple quantitative index would be very useful to describe the aggregation level of a bacterial population under different circumstances before deciding treatment methods or for studying signalling pathways.

In this study, we introduce an index, aggregation coefficient (AC), based on discrete Gini coefficient for the quantification of the aggregation patterns. Each 3D confocal image stack is divided into 3D sub-stacks, in which the biovolume is quantified. The biovolumes from each of the 3D image stacks are then sorted and calculated into one AC index number. When compared to the Gini coefficient, the sub-stacks are similar to different residential groups in a population, and the bacterial load in each unit is similar to the income of each group. As such, the inequality of the cell distribution in a population is quantifiable, describing, in a simple way, how compact or scattered are the aggregates, the method being suitable for high throughput comparison among different samples.

Our data showed that 12 out of 17 clinical isolates CF-PA formed much fewer surface-attached biofilms compared to the PAO1 strain when cultured in microtiter plates. In contrast, three of them, herein named PA08, PA37 and PA39, formed aggregates in M9 medium suspension with different sizes and characteristics. The AC index was applied to these and the numerical data was consistent with the biovolume distribution. Furthermore, we applied 250 μM Spermine NONOate as a nitric oxide donor, which was previously reported and confirmed to disperse *P. aeruginosa* PAO1 biofilms^27^, to the aggregates of PA08, PA37 and PA39. Among the three aggregating isolates, only PA37 showed dispersal with a reduction in aggregate sizes and proportions. Hence, our data not only provide a reliable and easy way for the description and comparison of different aggregation levels, but also delivers an important message that future drug tests on ‘biofilms’ should include both surface attached and floating consortiums, especially for CF *P. aeruginosa* isolates. Apart from clinical settings, AC may also be broadly applied to evaluate aggregates formed under a wide range of conditions, such as natural aquatic habitats^28,29^ and wastewater treatment^30^.

## Materials and Methods

### Ethics for cystic fibrosis patient sputum collection

Sputum samples from 72 patients with CF (median age at 21 years, range 17–62) were obtained by CF physiotherapist-assisted sample expectoration^31,32^ following Good Clinical Practice guidelines (ICH), with all sampling protocols and procedures approved by UK NHS Research Ethics Committee (South Central - Hampshire A Research Ethics Committee, Reference 08/H0502/126, Mechanisms of lung infection and inflammation in respiratory disease). Informed consent was obtained from all subjects or, if subjects were under 18, from a parent and/or legal guardian.

### Bacterial strains and culture conditions

*P. aeruginosa* PAO1 and strains isolated from CF sputum used in this study are listed in Table 1. Isolation of *P. aeruginosa* from sputa was carried out as previously described^33^. Routine overnight cultures were grown in lysogeny broth (LB) medium with shaking at 37 °C, 120 rpm for 15 hrs from a single colony on fresh overnight agar plates. For batch cultured *P. aeruginosa* biofilms, overnight cultures were diluted 1:100 into fresh M9, LB and BHI media to inoculate microtiter plates using 100 μl diluted culture. Microtiter plates were incubated statically with media changed every 24 hrs. Biofilms in microtiter plates were stained with 0.1% (w/v) crystal violet and dissolved in 30% (v/v) acetic acid. Crystal violet staining was determined at a wavelength of 584 nm. For aggregates, overnight cultures of *P. aeruginosa* were diluted 1:100 into standard M9 minimal medium containing 48 mM Na_2_HPO_4_, 22 mM KH_2_PO_4_, 9 mM NaCl, 19 mM NH_4_Cl, 2 mM MgSO_4_, 100 μM CaCl_2_, 20 mM glucose. 4 ml of diluted culture was inoculated into each well of 6 well plate and the plates were shaken at 50 rpm at 37 °C for 24 hrs. For NO treatment on aggregates, a final concentration of 250 μM S150 (Spermine NONOate, Sigma-Aldrich) was added to 22 hrs aggregate cultures and further incubated for another 2 hrs at 37 °C.

**Table 1:** *P. aeruginosa* strains used in this study.

Strains	Phenotypes^a^	Source or reference
PAO1	Wild-Type	^48^
PA08	CF-PA isolate	Sputum samples from 72 patients with CF (median age at informed consent 21 years, range 17–62; UK NHS Research Ethics Reference 08/H0502/126)
PA10	CF-PA isolate	
PA15	CF-PA isolate	
PA20	CF-PA isolate	
PA21	CF-PA isolate	
PA26	CF-PA isolate	
PA30	CF-PA isolate	
PA37	CF-PA isolate	
PA39	CF-PA isolate	
PA44	CF-PA isolate	
PA49	CF-PA isolate	
PA55	CF-PA isolate	
PA56	CF-PA isolate	
PA57	CF-PA isolate	
PA58	CF-PA isolate	
PA66	CF-PA isolate	
PA68	CF-PA isolate	

### Microscopic visualisation of *P. aeruginosa* aggregates

After 24 hrs incubation, the suspended aggregate culture in each well was transferred into a sterile universal tube. The tubes were gently inverted 5 times. For confocal laser scanning microscopic (CLSM) resulting 3D image stacks, 200 μl bacterial cultures were taken with a wide-cut 1 ml tip to avoid the shear force and stained with the LIVE/DEAD® BacLight Bacterial Viability Kit. Stained cultures were then gently transferred to tissue culture treated CELLview dishes (CELLview™ Greiner Bio-One) to facilitate the attachment of the samples. Stained samples were imaged with CLSM (Leica SP8) at a magnification of × 63. SYTO-9 was used for live cells and excited at 488 nm wavelength; propidium iodide was used as a dead cell marker and excited at 561 nm wavelength. This produced stacks of images of dimension 1024 × 1024 × (5–120,variate among samples) pixels and a voxel size of 0.241 × 0.241 × 0.488 μm.

### Fiji image processing

Processing of the 3D image stacks and quantification of the biovolume was done in Fiji (ImageJ 2.0/1.52n)^34^. All 3D image stacks were first converted to 8-bit and calibrated to the correct voxel size. Each calibrated 3D image stack was then divided into 3D sub-stack of 64 pixels × 64 pixels × 5 voxels by the LungJ^35^ plugin and placed in a folder. A custom-made Fiji macro (Supplementary Fiji macro 1) was then used to automatically process these sub-stacks. Briefly, for each sub-stack, the macro applied an automated Otsu thresholding algorithm to select the bacterial aggregates in each sub-stack and create a binary sub-stack. Then the macro used the 3D Manager function from the 3D ImageJ Suite plugin^36^ to find all 3D objects in the binary sub-stack, measure their volumes in (μm^3^), and export the data as a text file. For aggregate volumes calculation in each sample (micrograph), all 3D bacterial aggregates were detected and measured using Otsu and 3D Manager. For size distribution curves, different biovolumes were grouped into 26 categories ranging from 0 to 250000 μm^3^.

A second macro (Supplementary Fiji macro 2) was used to automatically calculate the total biovolume of the 3D bacterial aggregates from each text file (=sub-stack) and produce a summary text file. The biovolumes from all sub-stacks were then sorted and used for AC calculation.

### Mathematical formulation for aggregation coefficient

**Figure 1:**
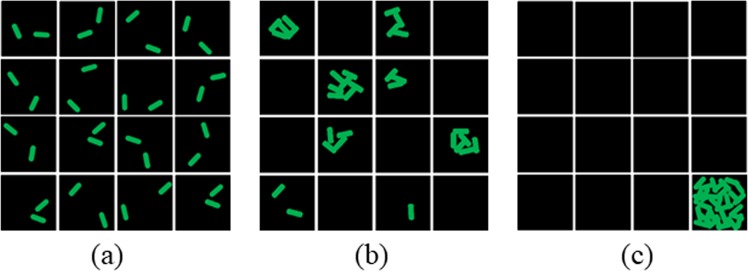
Schematic diagrams of different cell distributions in micrographs with the same total number of cells. (**a**) Cells distribute evenly (equidistribution), AC = 0. (**b**) Cells distribution is inhomogeneous, AC = 0.533 (**c**) Cells distribution is very concentrated, AC = 1.

**Figure 2:**
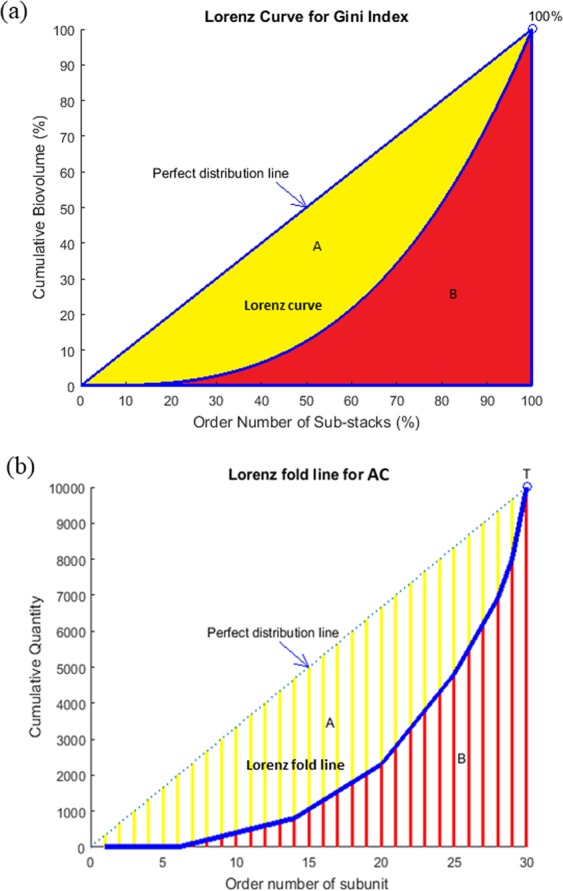
(**a**) Lorenz curve for a standard Gini coefficient (**b**) Modified Lorenz fold line for aggregation coefficient.

Various conditions may exist for different cell distributions and here we use three images in Fig. 1 as representatives. (a) shows a well-separated planktonic culture/bacterial suspension, AC = 0; (b) shows that under certain circumstances, such as nutrient/oxygen limitation, part of the bacterial community tends to form small aggregates, AC = 0.533; (c) genetically altered bacteria may produce excessive extracellular matrix that help the co-adhesion of the cells into a cluster, AC = 1. The distribution of cells within the defined sub-stacks in these 3 images are significantly different, which is similar to how the total income of a country is distributed into different groups of residents. However, a Lorenz curve is used in the Gini Index due to the continuous cumulative portions of population in Fig. 2a. In a stack of images, only a limited number of sub-stacks can be produced. Therefore, a variation, Lorenz fold line is used for AC calculation as shown in Fig. 2b. The order of sub-stacks was sorted based on the calculated biovolume of bacteria in each sub-stack, which would be the same as sorting the income residential groups from the lowest to the highest. The formulation of AC is described below using N = 30 sub-stacks as example:

1. Each stack of image is divided into N sub-stacks. The total volume in each sub-stack is defined as X_i_, where i = 1,2,3,…,N. Without losing generality, X_*i*_ values are rearranged into a sequence of values with non-decreasing order using computer program.
  $${{\rm{X}}}_{1}\le {{\rm{X}}}_{2}\le \ldots \,\ldots \le {{\rm{X}}}_{{\rm{N}}-1}\le {{\rm{X}}}_{{\rm{N}}}$$
2. Another variable - accumulative quantity Y_i_, is defined and calculated as
  $${{\rm{Y}}}_{{\rm{i}}}=\mathop{\sum }\limits_{k=1}^{i}\,{{\rm{X}}}_{{\rm{k}}}$$
  The total amount of all quantities in the image is T = Y_N_.
3. For most common situations, the distribution of cells in a 3D image stack is not perfectly equal, thus Y_i_ should locate below the diagonal line, generating the blue Lorenz fold line with discrete variables in Fig. 2b, which is comparable to the Lorenz curve with continuous variables in Fig. 2a. Similar to the calculation of area B below Lorenz curve, the Sum of all Accumulative Number Y_i_ (S) is introduced here, i.e.

$${\rm{S}}={{\rm{Y}}}_{1}+{{\rm{Y}}}_{2}+\ldots \,\ldots +{{\rm{Y}}}_{{\rm{N}}}$$

The maximum value of S occurs when each sub-stack contains the same amounts of cells, i.e. perfectly equal distribution.

$${{\rm{X}}}_{1}={{\rm{X}}}_{2}=\ldots \,\ldots ={{\rm{X}}}_{{\rm{N}}}={\rm{T}}/{\rm{N}},$$

then $${{\rm{Y}}}_{{\rm{i}}}={\rm{i}}\times \frac{T}{N}$$

S value reaches its maximum ${{\rm{S}}}_{{\rm{\max }}}=\frac{N\times (N+1)}{2}\times \frac{T}{N}=\frac{T\times (N+1)}{2}$

The minimum value of S occurs when 29 of the sub-stacks contain no cells and one sub-stack contains T cells, i.e, perfectly concentrated distribution.

$${{\rm{X}}}_{1}={{\rm{X}}}_{2}=\ldots \,\ldots ={{\rm{X}}}_{29}=0,\,{{\rm{X}}}_{30}={\rm{T}}$$

Then Y_1_ = Y_2_ = …… = Y_29_ = 0, Y_30_ = 100%T

As a result, S_min_ = T

When bacteria are randomly distributed, Y_i_ should locate at the Lorenz Fold Line and the AC value should be:

$${\rm{AC}}=\frac{[T\times \frac{(N+1)}{2}-S]}{[T\times \frac{(N+1)}{2}-T]}=\frac{(N+1-2\times \frac{S}{T})}{(N-1)}$$

Thus, 0 ≤ AC ≤ 1. When the cell number in each sub-stack is equal, AC = 0; when all the cells are concentrated in one sub-stack, AC = 1.

MATLAB is applied for calculating AC as follows:

%All quantities in each sub-stack have been assigned in vector Q beforehand.

$${\rm{Y}}={\rm{sort}}\,({\rm{Y}});\,{\rm{T}}=0;\,{\rm{S}}=0;\, \% \,{\rm{Rearrange}}\,{\rm{the}}\,{\rm{order}}\,{\rm{of}}\,{\rm{Quantity}}\,{\rm{of}}\,{\rm{every}}\,{\rm{sub}}-{\rm{stack}}.$$

$${\rm{for}}\,{\rm{i}}=1:{\rm{n}};\,{\rm{T}}={\rm{T}}+{\rm{Y}}({\rm{i}});\,{\rm{C}}({\rm{i}})={\rm{T}};\,{\rm{end}}\, \% {\rm{To}}\,{\rm{calculate}}\,{\rm{Cumulative}}\,{\rm{Quantiy}}\,\& \,{\rm{Total}}\,{\rm{amount}}.$$

$${\rm{for}}\,{\rm{i}}=1:{\rm{n}};\,{\rm{S}}={\rm{S}}+{\rm{C}}({\rm{i}});\,{\rm{end}}\, \% {\rm{To}}\,{\rm{calculate}}\,{\rm{the}}\,{\rm{value}}\,{\rm{S}}.$$

$${\rm{AC}}=({\rm{n}}+1-2\ast {\rm{S}}/{\rm{T}})/({\rm{n}}-1)\, \% {\rm{The}}\,{\rm{AC}}\,{\rm{value}}\,{\rm{is}}\,{\rm{obtained}}.$$

## Results

### CF-PA isolates are impaired in biofilm formation *in vitro*

**Figure 3:**
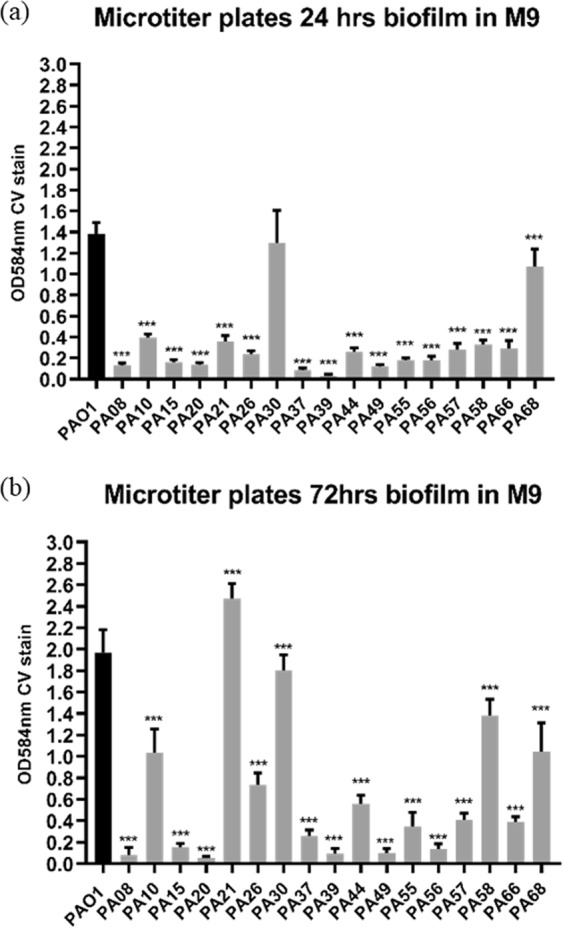
24 hrs (**a**) and 72 hrs (**b**) CF-PA isolates biofilms in M9 medium in microtiter plates. The biofilm formation of each CF-PA strain is compared to PAO1 using two-tailed Student T test. *** denotes P < 0.01, ** denotes 0.01 < P < 0.05. Data acquired from 3 independent experiments and 6 technical replicates.

17 CF-PA isolates were cultured for 24 and 72 hrs in M9, LB and BHI medium using standard microtiter plate assay due to the variance in growth rates. Two-tailed Student T test was applied to compare biofilm formation between PAO1 WT and each CF-PA isolate, with a confidence level set to 95%. Results showed that the biofilm formation ability of different strains showed a similar trend in M9, LB and BHI, but the biomass of PAO1, PA10, PA21, PA30, PA58 and PA68 was much higher in M9 than in LB or BHI after 72 hrs (Fig. 3 and Supplemental Figs. S1 and S2). Therefore, M9 promoted biofilm formation in batch culture and the comparison among different strains was carried out in M9 for this study. For 24 and 72 hrs culture, only PA30 showed relatively comparable biomass with PAO1 (P = 0.262 and 0.011, respectively), while all other CF-PA strains formed significantly different (mostly fewer) biofilms compared to PAO1 (P < 0.0001) as shown in Fig. 3, consistent with previous reports^37,38^.

### Some CF-PA preferably form suspended aggregates rather than attached biofilms

**Figure 4:**
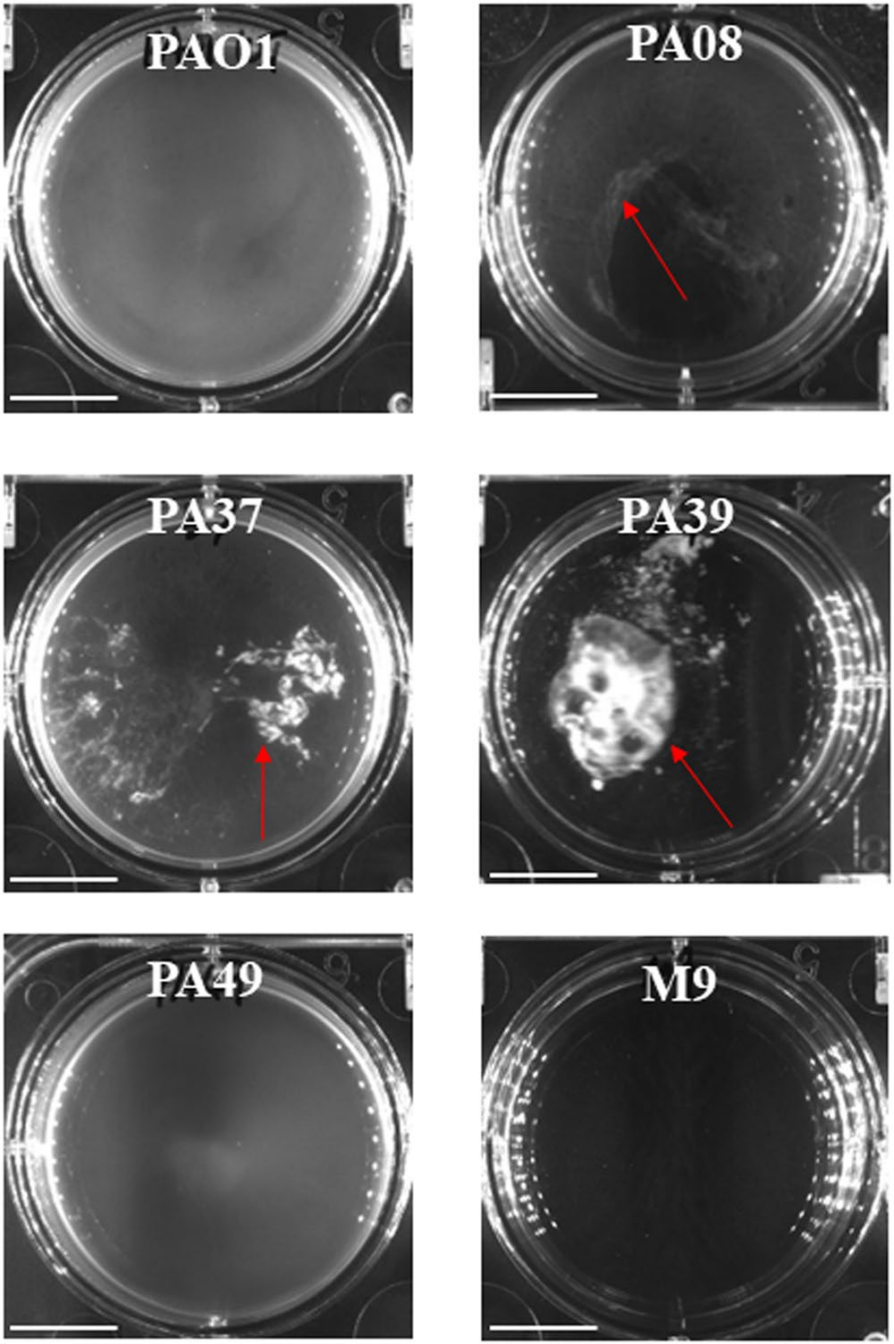
CF-PA suspended bacterial aggregates in M9 medium cultured in 6 well plates. Red arrows point at macro-aggregates. Scale bar = 1 cm.

We next investigated the morphologies of the planktonic cultures from these CF-PA isolates in M9 media. Among the strains with impaired biofilm formation, some formed visible aggregates in the suspended cultures (Fig. 4), which is consistent with previous reports claiming that nutrient depletion can lead to aggregation^16^. PA08, PA37 and PA39 showed significant aggregation independent of growth rate both in 6 well plate and under confocal microscopy, while all other strains showed no obvious aggregation (no particle over 30 μm^3^) and PA49 was chosen as the representative.

### AC index for planktonic aggregates

**Figure 5:**
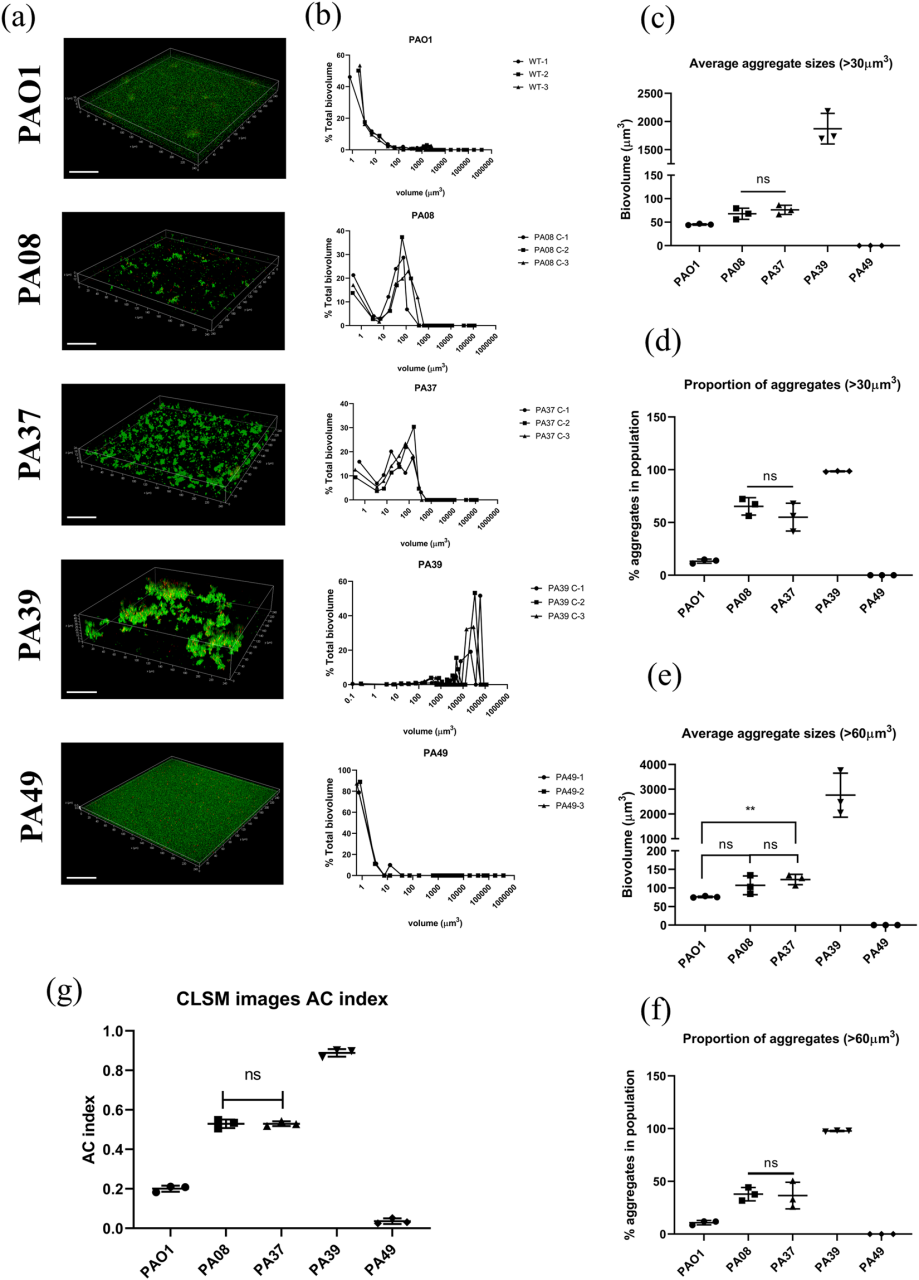
(**a**) The 3D micrographs of cell aggregates imaged by CLSM. Data acquired from 3 independent experiments. Scale bar = 50 µm. (**b**) Particle biovolumes distribution of different CF-PA planktonic cultures. The sizes of single cells and aggregates are grouped into 26 volume categories. The Sum and Mean biovolumes of particles in each category were calculated. For each category, the proportion of Sum biovolume in the total biovolume of entire micrograph is plotted to the Mean size. The details for each replicate was shown in the same graph to show the variances. (**c**) Average aggregate biovolume (>30 μm^3^) in each sample. (**d**) The proportion of all aggregates (>30 μm^3^) in the total biovolume of each sample. (**e**) Average aggregate biovolume (>60 μm^3^) in each sample. (**f**) The proportion of all aggregates (>60 μm^3^) in the total biovolume of each sample. (**g**) AC indexes of different CF-PA strains in planktonic cultures.

The 3D micrographs for planktonic CF-PA aggregates (Fig. 5a) were subjected to analysis. Four different algorithms were applied for the comparison of different aggregation levels in PAO1, PA08, PA37, PA39 and PA49 suspended cultures, including three prevalently used methods - particle size distribution curves^16^, average size^14,15^ and aggregates proportion^17^ (Fig. 5b–f), as well as the novel index AC (Fig. 5g). We defined two thresholds, over 30 μm^3^ and 60 μm^3^, as aggregates. Data showed that these five strains presented significantly different aggregation levels (One-way ANOVA and multiple tests, P < 0.0001), except for PA08 and PA37 which showed comparable levels using all four methods (Two-tailed Student T test, P > 0.7). The novel AC index was highly consistent with all three traditional methods. PA39 showed the largest aggregates in micrographs (Fig. 5a). The total biovolumes of PA39 suspended cultures were mainly (63.27 ± 9.09%, SD) large size particles (>10000 μm^3^) (Fig. 5b), showing the largest average aggregate sizes and the highest proportion of aggregates of PA39 (Fig. 5c–f). Accordingly, the AC indexes for PA39 cultures were the highest among all five strains (0.89 ± 0.02, SD) (Fig. 5g). In contrast, the total biovolumes of PA49 suspended culture were mainly (96.7 ± 5.72%, SD) composed of particles of 0–5 μm^3^ (Fig. 5b), with no visible aggregates in Figs. 4 and 5a. PA49 contained no particle over 30 μm^3^, therefore the aggregate sizes or proportions were 0 (Fig. 5c–f). Consistently, the lowest AC occurred in PA49 (0.036 ± 0.014, SD). PA37 and PA08 showed similar size distribution (Fig. 5b) where the populations were mainly contributed by particles volumes between 10–250 μm^3^, consistent with micrograph visualization despite the much lower total biovolume of PA08 than PA37 due a slower growth (data not shown). No significant difference was found for the average sizes or aggregate proportions of PA08 and PA37 cultures (Fig. 5c–f), and the AC indexes were consistently comparable (~0.53) (Fig. 5g). PAO1 cultures showed some aggregates but the majority were small particles, with the proportion of aggregates less than 15% and an average aggregate sizes of 43 ± 1.5 μm^3^ (30 μm^3^ threshold) (Fig. 5c,d), consistent with a previous report^16^. Accordingly, the AC indexes of PAO1 were higher than PA49 but much lower than other strains (0.201 ± 0.02, SD).

It is notable that when the threshold was defined as 60 μm^3^, the algorithm of average aggregate size lost its sensitivity to distinguish the difference between PAO1 and PA08 (Fig. 5e), indicating that the conclusion from average size may be threshold-dependent. AC index does not rely on thresholding, thus avoiding bias and misinterpretation of data. Taken together, AC can easily describe the relative aggregation trend of bacterial communities independent of the total biovolume, which is more accurate than just calculating and comparing absolute sizes when comparing strains with different growth rates.

### NO treatment for CF-PA isolates aggregates

**Figure 6:**
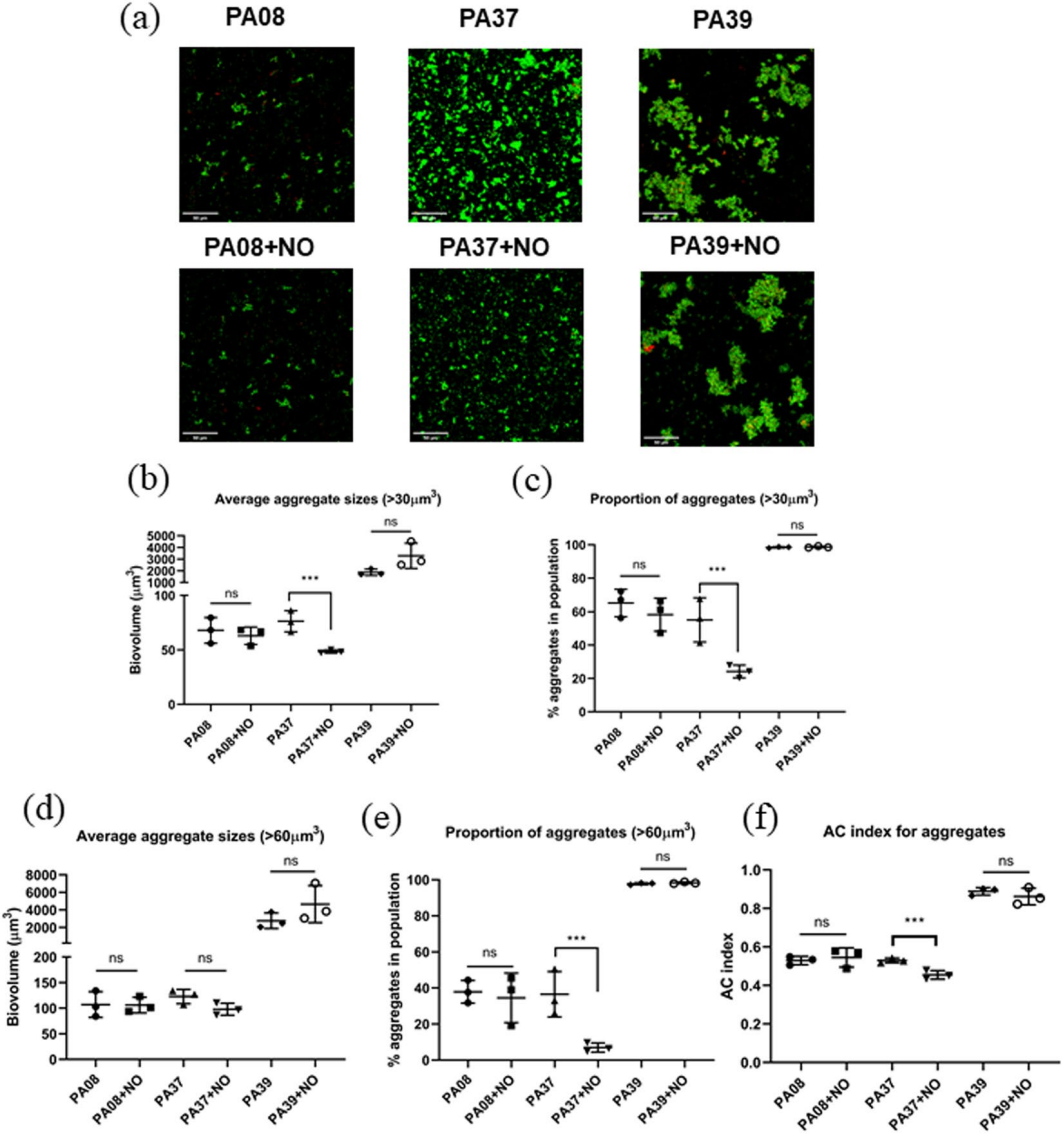
(**a**) The maximum projection micrographs of cell aggregates. Scale bar = 50 µm. (**b**) Average aggregate biovolume (>30 μm^3^) in each sample. (**c**) The proportion of all aggregates (>30 μm^3^) in the total biovolume of each sample. (**d**) Average aggregate biovolume (>60 μm^3^) in each sample. (**e**) The proportion of all aggregates (>60 μm^3^) in the total biovolume of each sample. (**f**) AC indexes of PA08, PA37 and PA39 suspended aggregates with and without NO treatment. *** denotes P < 0.01, ** denotes P < 0.05. Data acquired from 3 independent experiments.

Nitric oxide is known to disperse different *P. aeruginosa* biofilms^10,39^. A recent study^27^, as well as our preliminary data suggests that 250 μM Spermine NONOate (NO donor) is efficient in dispersing *P. aeruginosa* PAO1 and CF-PA isolates biofilms (data not shown). We proposed that NO may be able to disperse suspended aggregates as well, resulting in a reduction in aggregate sizes. Here we compared average aggregate sizes and proportion of aggregates with AC using NO treated PA08, PA37 and PA39 aggregates to test the sensitivity of different algorithms, as the comparison was carried out for the same strains with the same individual cell sizes. Two-tailed Student T test was applied to compare the aggregation levels before and after NO treatment. When the threshold of aggregate size was >30 μm^3^ as shown in Fig. 6b, the average aggregate sizes did not show significant change before and after NO treatment for PA08 (P = 0.953) and PA39 (P = 0.226), while PA37 showed a substantial reduction (P = 0.008), consistent with visualized results in Fig. 6a. Similarly, the reduction of aggregate proportions of these three strains (Fig. 6c) were consistent with the micrographs, showing dispersal effect only in PA37 (P = 0.018). However, when the aggregate was defined as biovolume over 60 μm^3^, average size method failed to reflect the dispersal of PA37, whilst aggregate proportion method still captured the effect (Fig. 6d,e). Therefore, the NO treatment data analysis again pointed at the potential bias of average size algorithms based on different thresholding. In contrast, the AC indexes are independent of thresholding process, showing that PA08 and PA39 population did not change after NO challenge in Fig. 6f (PA08 P = 0.634, PA39 P = 0.384), while PA37 AC index dropped significantly (P = 0.007). Hence, AC index data was further proven to be more reliable and objective when compared to average sizes, and the sensitivity of AC is sufficient to distinguish the changes in aggregation patterns before and after treatments.

## Discussion

Previous studies have found that *P. aeruginosa* is rarely localized to the airway epithelial cell surface but instead can be found abundantly localized in intraluminal material and imbedded in the mucus surrounded by PMNs (Polymorphonuclear neutrophils), filling the respiratory zone with aggregated bacteria^7,40,41^. In addition, bacterial aggregates are also frequently found in wound and urinary tract infections^5,42,43^. These embedded cell aggregations *in vivo* are regarded as biofilms not necessarily attached to a surface, with their actual volume and size being much smaller than *in vitro* biofilms^1^. Therefore, studies of non-attached aggregates are crucial to better understand *in vivo* bacterial aggregates, especially as it has been recognized recently that *in vitro* biofilm models are far from sufficient to uncover the reality of *in vivo* conditions^1^. Here we provide a new index to facilitate the description and measurement of non-attached aggregates using CF-PA isolates.

As shown in Figs. 3 and 4, the majority of CF-PA strains formed less biomass than PAO1, consistent with previous reports^37,38^. However, PA08, PA37 and PA39 are poor biofilm formers in microtiter plates, but mostly generated floating aggregates instead of attaching. Therefore, the bacterial communities of such strains can only be studied using suspended culture. Currently, the levels of cell aggregates have been mainly described in two ways: descriptive and quantitative. Direct microscopic or macroscopic images may be shown, where readers can compare the level of aggregation visually^13,18^. For quantification, some previously published research has used OD values for the measurement of aggregation. Low speed centrifugation can be used to separate cell aggregates and planktonic cells. The cell aggregates were then collected for vortex and CFU count so that the cell number that went into aggregate was calculated^44^. Alternatively, a static system was reported by comparing the OD value of suspension below the aggregating surfaces. With the same initial inoculum and growth rate, the lower the OD value of suspension (planktonic cells) the more aggregates^12^. However, these methods cannot directly reflect the characters of the cell aggregates themselves and are limited in the usage if strains with different growth rates are to be analysed. Modern equipment, such as flow cytometry and laser diffraction analysis (LDA) can be applied to determine the size of cell aggregates^16,17^. Similar methods include a cell counter to count individual cells within cell aggregates^45^. The counting results from these methods were presented in two ways^16,17^: (1) the percentages of aggregates in the total biovolume by defining a size threshold for the determination of aggregates; (2) grouping cells of different size ranges into different categories and calculate the proportion of each category in total biovolume. A simple aggregates percentage for each population is easy for comparison but does not show if the population is mainly composed of a few large aggregates or many small aggregates, i.e., aggregation pattern. In contrast, plotting the proportion of each size category gives relative detailed information on the actual size distribution of aggregates. However, this method results in different graphs for different populations, making it hard to describe large scale samples. Furthermore, comparing the absolute values of strains with dramatic differences in individual cell size or multispecies aggregates is inaccurate. Under the circumstances where samples cannot be easily subjected to LDA or flow cytometers, analysis from micrographs is a simpler option. Some studies directly measured the sizes of aggregates based on pixels or areas using ImageJ^14,15^. The means and standard deviations of the aggregate sizes were calculated to show the differences in the level of cell aggregations. Similarly, it is difficult to conclude the aggregation levels of two populations with only the comparable mean sizes but very different standard deviations, and the definition of threshold is subjective. In this study, we introduce a variation of the Gini coefficient for quantifying bacterial aggregation, herein named the Aggregation Coefficient (AC), and compared this with the size distribution plotting methods mentioned above. AC only gives out one number for each micrograph from a sample and can directly reflect the level of cell aggregation. As shown in Fig. 5, the calculation of AC aligned well with both visual judgement and three prevalently used algorithms for aggregation level description. The growth rates of PA08 were slower than PA37, but within different total biovolumes, the similar size aggregates (50–100 μm^3^) constituted the highest proportion (~40%). Consistently, the AC coefficient of these two strains were comparable, indicating the same aggregation patterns. Thus, AC provides an easy way to measure the level of cell aggregation, especially to distinguish samples that possess small but significantly different levels of aggregation.

As nitric oxide has been repeatedly reported to disperse *in vitro P. aeruginosa* biofilms^10,27,39,46,47^, particularly with a recent study showing its effect in CF patient^33^, a commercially available NO donor S150 was applied in this study to disperse non-attached CF-PA aggregates. When individual aggregates were defined as connected biovolume >30 μm^3^, PA37 aggregates dispersal was reflected successfully by frequently adopted methods, with a ~36% reduction in mean aggregate size (P = 0.0082) and ~80% reduction of aggregate proportion (P = 0.0176) (Fig. 6b,c). In contrast, PA08 and PA39 showed tolerance to NO using these two calculation methods (Fig. 6b,c). These data are consistent with micrographs shown in Fig. 6a. Whilst the same conclusion can be obtained with an aggregate size threshold of 40 and 50 μm^3^ (data not shown), the method of mean aggregate size failed to capture the dispersal effect from PA37 when aggregates are defined as biovolume >60 μm^3^ (Fig. 6d,e). However, AC algorithm successfully reflected the dispersal of PA37 aggregates (P = 0.007) as well as the tolerance of PA08 and PA39 (Fig. 6f) and does not rely on subjective size thresholding. Therefore, AC can be applied more accurately to avoid false positive or negative data interpretation.

Overall, this novel application of AC not only successfully distinguishes different aggregation levels of different CF-PA strains, but also shows that NO treatments that are effective towards surface attached biofilms may or may not be sufficient to disperse suspended aggregates. For biofilm drug tolerance tests in the future, both attached and suspended cultures should be considered, as they might have different physiological properties for resistance/tolerance. The results of AC calculation are consistent with both visualisation and the traditional plotted size distribution curves, but much easier to quantify and compare with statistics. Furthermore, the AC calculation is based on 3D micrographs, which is widely accessible in many laboratories. The Fiji plugin macro and MATLAB coding are provided here so that the formula can be easily applied by other researchers.

## Supplementary information

Supplementary information

## Data Availability

Fiji macro codes are available in Supplementary Information Files.
