# Supplementary material for: A novel application of Gini coefficient for the quantitative measurement of bacterial aggregation
Source: Sci Rep. 2019 Dec 12;9:19002. doi: 10.1038/s41598-019-55567-z (PMC6908595; doi:10.1038/s41598-019-55567-z)
Supplement: Supplementary file 1 — Supplementary information [file 41598_2019_55567_MOESM1_ESM.pdf]

Supplement to:

A novel application of Gini coefficient for the quantitative measurement of bacterial aggregation

Yu-Ming Cai[a], David S. Chatelet[b], Robert P. Howlin[c], Zhi-Zhong Wang[d], Jeremy S. Webb[a*]

[a]National Biofilms Innovation Centre, Institute of Life Sciences, University of Southampton, Southampton, SO17 1BJ, UK

[b]Biomedical Imaging Unit, University of Southampton, Southampton, SO16 6YD, UK

[c]NIHR Southampton Respiratory Biomedical Research Centre, Southampton SO16 6YD, UK

[d]School of Biomedical Engineering, Shanghai Jiao Tong University, 800 Dongchuan Rd, Minhang District, Shanghai, P.R.C

Supplementary Fiji macro 1: Biovolume calculation in each divided sub-stack within defined folders

```
dir1 = getDirectory("Choose source folder");
dir2 = getDirectory("Create and select a folder for .txt results");
dir3 = getDirectory("Create and select a folder for binary images");
listAll = getFileList(dir1);

setBatchMode(true);

listImage = newArray();
for(i = 0; i < listAll.length; i++){
	if(endsWith(listAll[i], ".tif")){
		listImage = Array.concat(listImage, listAll[i]);
	}
}
Array.sort(listImage);

for(i=0; i<listImage.length; i++){
	open(dir1+listImage[i]);
	cubeTitle = getTitle();
	run("8-bit");

	run("Duplicate...", "title=B duplicate");
	duplicateTitle = getTitle();
	run("Set Measurements...", "area limit display redirect=None decimal=3");
```

```
	selectWindow(duplicateTitle);
	NbSlice = nSlices;
	for(n = 1; n <= NbSlice; n++){
		setSlice(n);
		setAutoThreshold("Otsu dark no-reset");
		//run("Threshold...");
		setAutoThreshold("Otsu dark no-reset");
		run("Measure");
	}
	selectWindow(duplicateTitle);
	close();
	selectWindow("Results");
	for(n = 0; n<nResults; n++){
		resultArea = getResult("Area", n);
		if(resultArea > 0){
			Object3D = 1;
		}
		else {
			Object3D = 0;
		}
	}

	if(Object3D == 0){
		selectWindow("Results");
		saveAs("Text", dir2+"M_"+cubeTitle+"_Results.txt");
		run("Close");
		print(cubeTitle+": no 3D objects found");
		print("-------------");
	}
	else {
		selectWindow("Results");
		run("Close");
	}

	if(Object3D == 1){
		selectWindow(cubeTitle);

		setAutoThreshold("Otsu dark no-reset");
		//run("Threshold...");
		setOption("BlackBackground", false);
		run("Convert to Mask", "method=Otsu background=Dark calculate");
		saveAs("Tif", dir3+cubeTitle+"_binary.tif");

		// some options in Manager3D Options, select Plugins/Record to record
		run("3D Manager Options", "volume sync
distance_between_centers=10distance_max_contact=1.80");
		// run te manager 3D and add image
		run("3D Manager");
		// segment the signal at a defined threshold range
```

```
		Ext.Manager3D_Segment(250, 255);
		// add the segmented signal (3D objects) to ROI manager
		Ext.Manager3D_AddImage();
		// Measure the Volume of 3D objects and save as csv file
		Ext.Manager3D_Measure();
		Ext.Manager3D_SaveResult("M", dir2+cubeTitle+"_Results3D.txt");
		// close the result window and 3D Manager
		Ext.Manager3D_CloseResult("M");
		//Ext.Manager3D_Reset();
		Ext.Manager3D_Close();

		run("Close All");
		print("-------------");
	}
}
print("All images have been processed!");
selectWindow("Log");
saveAs("Text", dir2+"Log.txt");
run("Close");
exit("ALL IMAGES HAVE BEEN PROCESSED");
```

Supplementary Fiji macro 2. Fiji macro 2 for extracting 'volume' data from txt result files of all sub-stacks into a summary file.

```
dir2 = getDirectory("Select folder with results files");
folderName = File.getName(dir2);
listTxt = getFileList(dir2);

listResults = newArray();
for(i=0; i<listTxt.length; i++){
	if(endsWith(listTxt[i], "_Results.txt") | endsWith(listTxt[i],
"_Results3D.txt")){
		listResults = Array.concat(listResults, listTxt[i]);
	}
}
Array.sort(listResults);

volumeTot_array = newArray();

for(i=0; i<listResults.length; i++){
	filePath = dir2+listResults[i];
	run("Results... ", "open=filePath");

	valueTot = 0;
```

```
		if(endsWith(listResults[i], "Results.txt")){
			for(n=0; n<nResults; n++){
				value = getResult("Area", n);
				valueTot = valueTot + value;
			}
		}
		else {
			for(n=0; n<nResults; n++){
				value = getResult("Vol (unit)", n);
				valueTot = valueTot + value;
			}
		}
		volumeTot_array = Array.concat(volumeTot_array, valueTot);
		run("Close");
}

if (nResults>=0){
		run("Clear Results");
}
i = nResults;

for(i=0; i<volumeTot_array.length; i++){
		setResult("Cube", i, listResults[i]);
		setResult("Volume total", i, volumeTot_array[i]);
}
updateResults;
saveAs("Results", dir2+folderName+"_Results.txt");
run("Close");
```

(a)

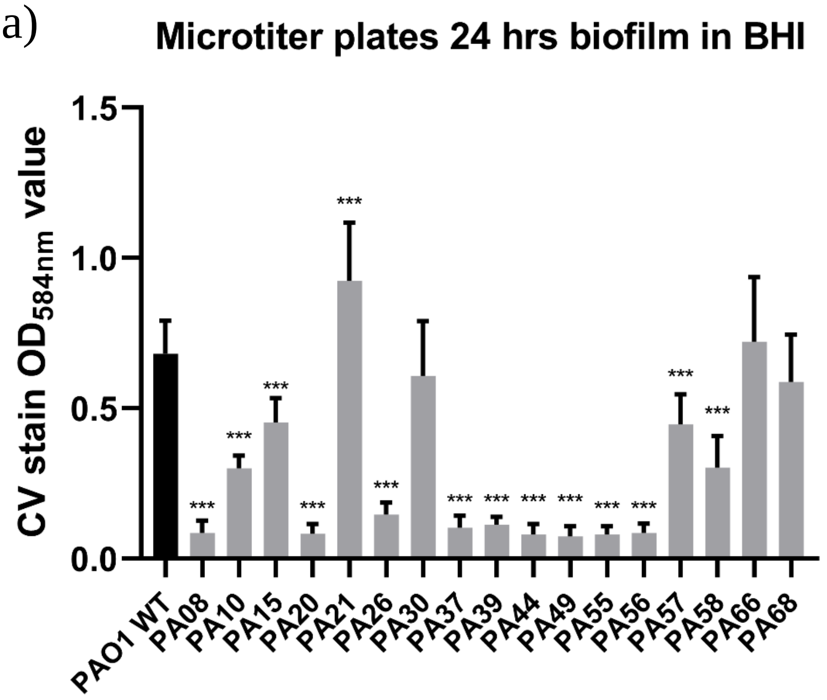

(b)

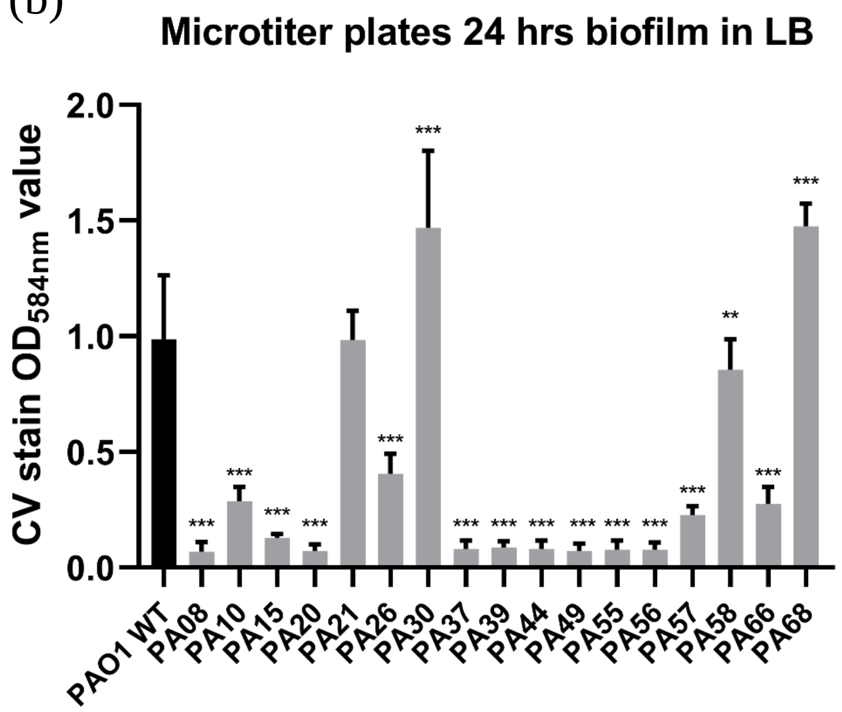

Supplemental Figure S1. 24 hrs CF-PA isolates biofilms in BHI (a) and LB (b) medium in microtiter plates. The biofilm formation of each CF-PA strain is compared to PAO1 using two-tailed Student T test. *** denotes $P<0.01$, ** denotes $0.01<P<0.05$. Data acquired from 3 independent experiments and 6 technical replicates.

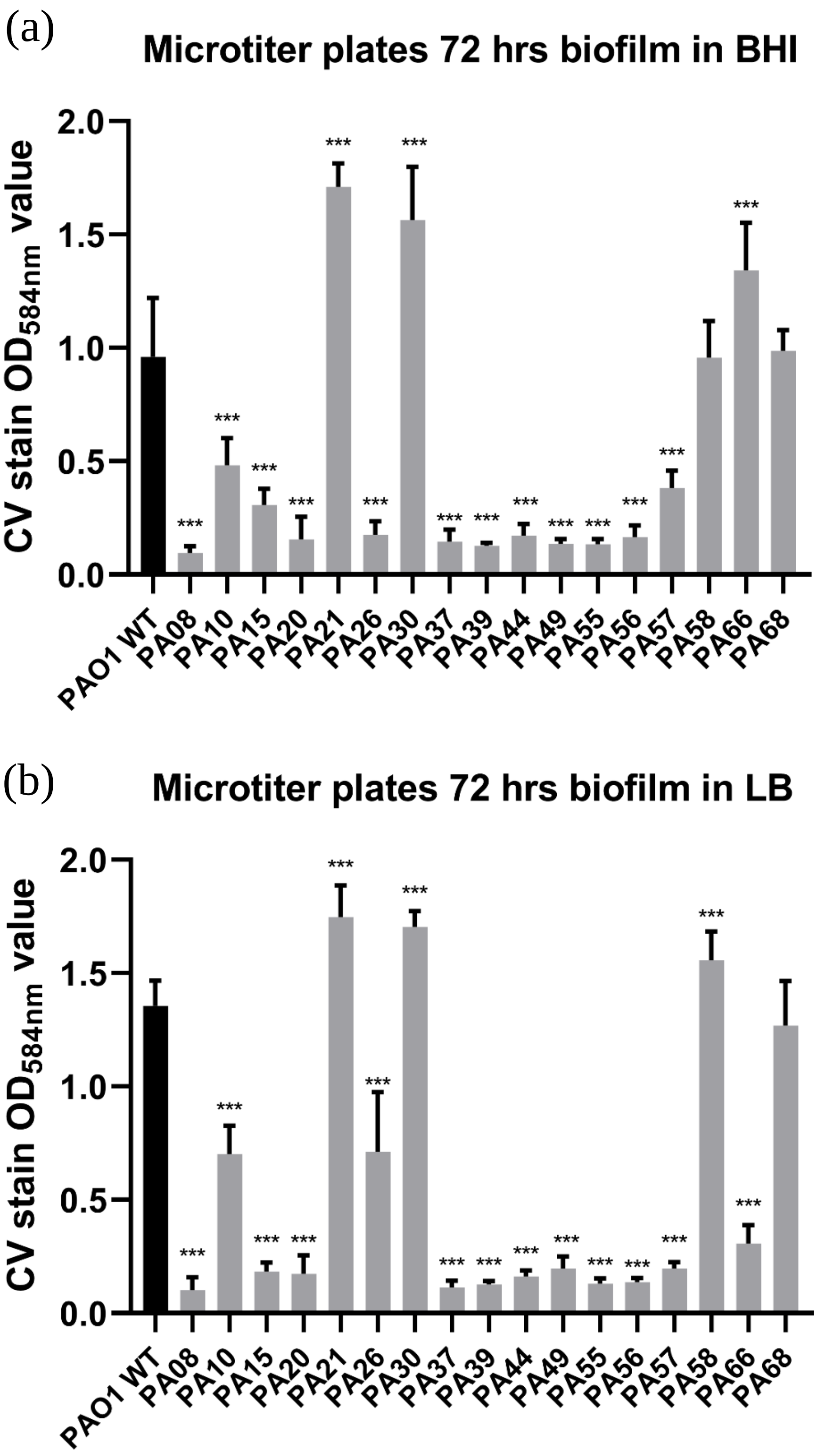

Supplemental Figure S2. 72 hrs CF-PA isolates biofilms in BHI (a) and LB (b) medium in microtiter plates. The biofilm formation of each CF-PA strain is compared to PAO1 using two-tailed Student T test. *** denotes P<0.01, ** denotes 0.01<P<0.05. Data acquired from 3 independent experiments and 6 technical replicates.
